# Cardiovascular Activity of the Chemical Constituents of Essential Oils

**DOI:** 10.3390/molecules22091539

**Published:** 2017-09-17

**Authors:** Tadeu Uggere de Andrade, Girlandia Alexandre Brasil, Denise Coutinho Endringer, Flávio Rogério da Nóbrega, Damião Pergentino de Sousa

**Affiliations:** 1 Departamento de Farmácia, Universidade de Vila Velha, Vila Velha ES 29102-770, Brazil; tadeu.uggere@uvv.br (T.U.d.A.); girlandiabrasil@hotmail.com (G.A.B.); denise.endringer@uvv.br (D.C.E.); 2 Departamento de Ciências Farmacêuticas, Universidade Federal da Paraíba, João Pessoa PB 58051-970, Brazil; frnobrega@hotmail.com

**Keywords:** monoterpene, sesquiterpene, phenylpropanoid, natural products, spasmolytic, smooth muscle, hypertension, heart, vasorelaxation

## Abstract

Cardiovascular diseases are a leading cause of death in developed and developing countries and decrease the quality of life, which has enormous social and economic consequences for the population. Recent studies on essential oils have attracted attention and encouraged continued research of this group of natural products because of their effects on the cardiovascular system. The pharmacological data indicate a therapeutic potential for essential oils for use in the treatment of cardiovascular diseases. Therefore, this review reports the current studies of essential oils chemical constituents with cardiovascular activity, including a description of their mechanisms of action.

## 1. Introduction

Non-communicable diseases (NCD) are the leading causes of death worldwide and are responsible for approximately 68% of all global deaths in 2012. The main causes for NCD are cardiovascular diseases (CVD), which account for 46.2% of NCD-related deaths. CVD are responsible for 17.3 million deaths per year, a number that is expected to grow to more than 23.6 million by 2030 [1]. Although a wide range of pharmacological agents are available for the treatment of CVD, the control and prevention of these diseases continues to be a challenge. The cost of treatment continues to increase, which makes CVD even more expensive and impactful to the budget of public health systems around the world [2]. Therefore, new tools for the treatment of CVD are necessary for a cost that is reasonable for health systems. Among the sources of drug candidates available for the treatment of CVD, natural products have been reported to have a potential role in the therapeutic effects of such diseases [3]. The evaluation of the chemical constituents of plants has enriched the pharmacological arsenal used in the treatment of diseases, and it has helped to understand their pathological mechanisms [4].

It is well established that oxidative stress influences the pathogenesis of heart diseases, such as hypertension, atrial fibrillation, and atherosclerosis, and studies have demonstrated the implication of this stress in these diseases [5]. There is substantial epidemiological and experimental evidence that antioxidants in the diet can be preventive for heart disease [5]. The failure of antioxidants found in foods, such as vitamins C and E, to prevent these disorders has led to the exploration of the ROS-suppressive effects of drugs used in the treatment of cardiovascular disease [6,7]. Moreover, many natural products have antioxidant and various other pharmacological properties [8]. Some of their actions on oxidative stress are associated with pharmacological effects [9,10,11,12]. Natural products with antioxidant and/or vasorelaxant effects have been shown to positively affect many cardiovascular conditions [13,14] once the redox status or the vascular function has worsened and is implicated in the pathogenesis of the CVD [15,16]. Several studies have shown that the antioxidant properties of natural products have potential uses in the prevention and treatment of some heart diseases [5,17,18]. One of the natural products that have piqued the current interest of researchers are essential oils [19]. This group of natural products consists of aromatic and lipophilic substances that are found in many medicinal plants. At present, over 3000 essential oils are known, of which 300 are commercially important, especially in the pharmaceutical, food, sanitary, cosmetic, and perfume industries [20]. Recent reviews have shown the therapeutic potential of this group in multiple areas, including: analgesics [21,22], anticonvulsants [23], anti-inflammatories [24,25,26], anticancer agents [27,28,29], anxiolytics [30], and antiulcer agents [31]. Essential oils and their constituents have also been shown to be promising substances in the development of therapeutic agents applied to cardiovascular diseases. These findings not only support the traditional use of aromatic plants and their essential oils but also highlight new cardiovascular functions of these natural products that were previously unknown [32]. Therefore, the purpose of this review was to conduct a systematic investigation of essential oil studies in experimental models related to cardiovascular activity.

The search was conducted in the scientific database PubMed, focusing on works published during the last five years (January 2011 to December 2015). The data were selected using the following terms: “essential oils”, “monoterpene”, and “cardiovascular”.

## 2. Results and Discussion

### 2.1. Thymoquinone

Thymoquinone is the main monoterpene of the volatile oil obtained from the seeds of *Nigella sativa* L. (Ranunculaceae). It exhibits several pharmacological activities, such as acting as an anti-inflammatory [26], antitumor agent [29] and as an analgesic [21]. Antioxidant and vascular relaxant effects of thymoquinone (TQ) have been documented in different experimental models of cardiovascular diseases. Aydin et al. [33] observed that treatment with TQ was associated with the reduction in the oxidative stress systemically measured after abdominal aorta ischemia or reperfusion injury in rats. This reduction in oxidative stress was associated with less severe lesions in the hearts of rats that received TQ intraperitoneally. The antioxidant mechanism of TQ involves reactive oxygen species (ROS), redox-related enzymes, and cytokine profiles. Nemmar et al. [34] observed that TQ improved the superoxide dismutase activity and reduced the interleucin-6 content in mice, which prevented cardiovascular side effects induced by a pollutant. TQ induced a dose-dependent reduction in plasmatic lactate dehydrogenase (LDH), thiobarbituric acid reactive substances (TBARS), and glutathione reductase (GR), whereas SOD activity in the plasma and the myocardial reduced or oxidized glutathione ratio (GSH/GSSG) were increased in rats with an isoproterenol-induced myocardial infarction that were then treated with TQ. These actions protect the heart from the injury resulting from isoproterenol [35]. Oxidative stress is also implicated in vascular dysfunction by blunting both nitric oxide (NO)- and endothelium-derived hyperpolarizing factor (EDHF)-mediated relaxation in the arteries of ageing animals. TQ could reverse the age-related down-regulation of the endothelial NO synthase (eNOS) and increased the vascular formation of ROS in the mesenteric artery. In addition to the effects on oxidative stress, TQ also affects components of the renin-angiotensin aldosterone system (RAS) and increases the expression of the small and intermediate conductance Ca^2+^-activated K^+^ channel (SKCa and IKCa, respectively), resulting in the incremental NO- and EDFH-induced mesenteric relaxation. These actions determined the improvement of endothelial function in ageing animals after 14 days of TQ treatment [36]. In addition, Ghayur et al. [37] observed that the relaxant effect of TQ in the aortas of rats should also involve the blockade of voltage-operated Ca^2+^ channels (VOCC). Taken together, these studies indicate that TQ has broad actions on the cardiovascular system, including the redox system, ion channels, the RAS system, endothelial-related relaxant agents, and cytokines. These actions result in antioxidant and vascular relaxant effects which work to protect the cardiovascular system.

### 2.2. Cinnamaldehyde

This aromatic aldehyde is an active constituent isolated from the stem bark of cinnamon trees such as *Cinnamomum cassia* Presl. and others plants [38]. El-Bassossy et al. [39] showed that cinnamaldehyde normalized the vascular contractility and prevented the development of hypertension in insulin deficient or resistant animals, also prevented the hyper-responsiveness to vasoconstrictor agents (Phenylephrine or KCl) and the hypo-responsiveness to vasodilatory agents (Ach) in the aortic rings. In insulin-deficient animals, cinnamaldehyde prevented the inhibition of NO release, while in insulin resistant rats, cinnamaldehyde prevented the elevated influx of Ca^2+^. Through an antioxidant action and the preservation of the NO levels, cinnamaldehyde protected the endothelium relaxation in the aortic rings of hyperglycemic mice. This antioxidant mechanism involved the up-regulation of the endogenous antioxidant enzyme NF-E2-related factor 2 (Nrf2) [40], which is known to regulate the generation of ROS [41]. Additionally, Raffai et al. [42] demonstrated that cinnamaldehyde also induced vascular relaxation in porcine coronary arteries through an endothelium-independent mechanism by inhibiting Ca^2+^ sensitivity and Ca^2+^ influx. When carried in micelles, cinnamaldehyde was also able to induce endothelium-dependent vascular relaxation by NO- and H_2_O_2_-dependent means. This was also demonstrated by Xue et al. [43], who showed that this relaxant effect of cinnamaldehyde was likely mediated by changes in calcium influx or in its release from intracellular stores. Additionally, Alvarez-Collazo et al. [44] studied the mechanism of action of this compound and showed that CA promoted relaxation in vascular smooth muscle cells (VSMC) and ventricular cardiac myocytes (VCM), at least in part by inhibition of the L-Type Ca^2+^ channel. Additionally, these studies do not exclude subtype 1 of the Transient Receptor Potential Ankyrin (TRPA1).

Song et al. [14] evaluated the cardioprotective properties of cinnamaldehyde against the ischemic injury precipitated by isoproterenol. The pre-treatment for 14 days with cinnamaldehyde resulted in decreased cardiac injury (hypertrophy and histological changes), decreased pro-inflammatory cytokines (TNF-α and IL-6), increased serum NO and SOD levels on the heart, and reduced ST segments generated by myocardial ischemia. Together, these results show that cinnamaldehyde has cardioprotective effects that can be attributed to antioxidant and anti-inflammatory proprieties.

### 2.3. Cinnamic Acid

This compound is an aromatic carboxylic acid with carbonic structure C6–C3 appearing naturally in the plant kingdom. In addition, cinnamic acids are formed in the biosynthetic pathway leading to various natural product classes, such as phenylpropanoids, flavonoids, coumarins, lignans, anthocyanins, and tannins [45]. *In vitro* analysis of vasorelaxant proprieties of cinnamic acid (CA) was conducted by Kang et al. [46] who used the thoracic aortas of rats. They showed that CA promoted vasorelaxation in a dose- and endothelium-dependent manner, once both NO inhibition by l-NAME and endothelium removing reduced its relaxant effect. The increases of e-NOS and pe-NOS after CA incubation on the cell cultures of human endothelium strongly reinforced this hypothesis. Additionally, the participation of Protein Kinase G (PKG) as a mediator was demonstrated due to the increase of cGMP observed after the incubation of cells with CA. Together, these results demonstrate that CA promotes vasorelaxant effects by changing the endothelium-NO release mechanism. Lastly, the mediation of intracellular Ca^2+^ release by PKG could be involved with those observations. Song et al. [14] studied the effects of CA on myocardial injury (MI), using an *in vivo* model. CA treatment deceased the biochemical (CK-MB and LDH) and inflammatory markers of MI (TNF-α and IL-6) and increased the NO levels, which resulted in the reduction of cardiac histological abnormalities induced by isoproterenol. These benefits were related, at least in part, to the improvement of NO synthesis and the antioxidant effect of this compound.

### 2.4. Cinnamyl Alcohol

Cinnamyl alcohol is a phenylproppanoid that occurs in numerous natural products in free state or as an ester in plants, such as cinnamon leaf, narcissus, and gardenia. This compound can also be obtained by the hydrogenation of cinnamic aldehyde. The vasodilatory propriety of cinnamyl alcohol (CAL) was evaluated by Kang et al. [47]. They used aortic rings, the cell cultures of human aortic smooth muscle cells and human umbilical vein endothelial cells. The results on the aortic rings showed an endothelium-dependent vasodilation. This effect was reduced by l-NAME, glibenclamide and methylene blue pre-treatment, which indicated the role of NO, K^+^ channel and guanylylcyclase, respectively, on the vasodilatory activity of CAL. It was also demonstrated that CAL promoted cGMP accumulation and the augmentation of PKG1 levels, and interfered with the Rho-kinase pathway. Therefore, the endothelium-dependent vasodilation induced by CAL is related with the NO-cGMP-PKG pathway in rat thoracic aorta, resulting in activation of K^+^ channels and an inhibition of the Rho-kinase pathway.

### 2.5. α-Bisabolol

α-Bisabolol is a monocyclic sesquiterpene tertiary alcohol, which has a weak sweet, floral aroma. It is found in substantial amounts in the essential oils of *Matricaria chamomilla*, *Salvia runcinata*, *Eremanthus erythropappus, Myoporum grassifolium*, and *Vanillosmopsis* sp [48]. Studies that evaluated the vasorelaxant proprieties of (−)-α-bisabolol (BS) were concentrated on the aortic and mesenteric arteries. De Siqueira et al. [49] observed the relaxant action of BS in a wide range of smooth muscle preparations, with a higher pharmacological potency in the mesenteric vessels. Pre-contracted aortic rings also showed relaxation with BS administration. Later, the same group demonstrated the BS-induced vascular relaxation mechanism involved with the calcium influx through voltage-dependent channels [50]. Calcium-dependent vascular relaxation was also observed in porcine coronary and splenic arteries once the withdrawal of the calcium from the medium completely eliminated the BS-induced vascular relaxation [51]. Therefore, the studies that were conducted have demonstrated that BS promoted vasorelaxation by inhibiting the calcium influx through voltage-dependent channels. The evaluation of the molecular model of the β-subunit isoform of voltage-gated L-type Ca^2+^ channel (Cavβ2a) demonstrated that BS interacted preferentially with this channel subunit and promoted the uncoupling of the Cavβ2a subunit from the α-interaction domain (AID). However, the authors do not exclude that BS could have also acted as a negative allosteric inhibitor.

### 2.6. Carvacrol

Carvacrol is a monoterpene phenol found in many essential oils. The compound is an isomer of thymol. Shabir et al. [52] observed that carvacrol decreased the hypercontraction induced by lead (Pb II) in the aortic rings of rats at a concentration of 100 μmol/L. The incubation of carvacrol with apocynin, which is an inhibitor of NADPH oxidase enzyme, did not promote any change in the vasodilatory response. However, co-incubation with l-NAME decreased the effect of carvacrol, which indicated that the relaxant effect of this compound was mediated by an increase in NO synthesis. Pires et al. [13] used cell cultures from parenchymal arterioles and tested the effect of carvacrol on calcium permeability on the transient receptor potential vanilloid (TRPV). The authors showed that carvacrol promoted an increased influx of calcium by activating the TRPV3 channel. Therefore, this calcium influx through the TRPV3 channel, which is important to activate intermediate (IK) and small-conductance Ca^2+^-activated K^+^ (SK) channels and causes hyperpolarization, may contribute to carbachol-endothelium dependent vascular relaxation. *In vivo* studies conducted by Dantas et al. [53] demonstrated that carvacrol induced hypotension and bradycardia in non-anesthetized Wistar rats. The mechanism apparently involved vasorelaxation through the reduction of Ca^2+^ influx by changes in voltage-dependent, transient receptor potentials and store/receptor operator channels (SOCs/ROCs). Taken together, these studies demonstrate that Carvacrol can cause vascular relaxation by an endothelium-dependent mechanism that involves the NO and Ca^2+^ pathways.

### 2.7. Borneol

Borneol is a cyclic monoterpene alcohol extracted from *Cinnamomum camphora* (L.) and other plants [54]. Silva-Filho et al. [55] demonstrated that borneol promoted the relaxation of aortic rings that were pre-contracted with phenylephrine or KCl^−^ in a concentration-dependent and endothelium-independent manner. Additionally, the pre-incubation with K^+^ channel blockers attenuated the borneol-induced vasorelaxation. Borneol also interfered with intracellular calcium mobilization. Bai et al. [56] observed that a borneol-rich extract at a concentration of 1 mg/mL promoted relaxation, with total relaxation that was obtained at 10 mg/mL. It was observed that a *Suxiao Jiuxin* Pill was able to promote vasorelaxation by both endothelium-dependent and -independent mechanisms. Wu et al. [57] conducted an *in vivo* study to determine if borneol had neuroprotective properties against ischemic stroke. The anti-inflammatory proprieties of borneol were demonstrated in this study. Borneol alone (0.8 mg/kg) was able to promote a reduction on the protein expression of pro-inflammatory markers (TNF-α, iNOS, IL-1β and COX-2). Additionally, borneol promoted a reduction of the infarct area in a dose-dependent manner (IC_50_: 0.36 mg/kg). Together, these results demonstrated the neuroprotective effects of borneol and promoted an indirect increase of the scavengers of ROS, and once the expression of iNOS was reduced the production of NO decreased.

### 2.8. Carvone

Carvone is a monoterpene ketone and the main active component of the oil of *Mentha spicata*. The carvones ((+)- and (−)-forms) are probably the most versatile terpene chirogens and suitable starting materials in stereoselective synthesis, especially terpenes [58]. The most well-known source of (−)-(*R*)-carvone is spearmint oil. Its enantiomer is a constituent of dill and caraway oils [59]. To investigate the effect of carvone on conductance arteries, Kundu et al. [60] demonstrated that this terpenoid was capable of promoting vasorelaxation on pre-contracted aortic rings, even when the artery was exposed to metals (arsenic and mercury). The antioxidant proprieties of carvone contributed to this vasorelaxant effect. However, the effect of carvone on calcium voltage-dependent channels was more important than its ROS scavenger or NO synthesis actions. Using aortic rings and guinea pig tracheas, De Sousa et al. [61] showed that there was no difference in the pharmacological action of (+)- and (−)-enantiomers of carvone, both forms presented a vasorelaxant action. Furthermore, it seems that the action was directly on the smooth muscle, since it was not reduced in the endothelium deprived rings.

### 2.9. Eugenol

This phenylpropanoid is used as an ingredient in cosmetics, perfumes, and pharmaceutical and dental preparations [62]. Kundu et al. [60] demonstrated that eugenol presented antioxidant proprieties and could promote a vasorelaxant effect, and a calcium blockade in high concentrations. The protective effect was observed in arteries that were exposed to heavy metal (As and Hg). Along the same line, Shabir et al. [52] examined that the effects of Eugenol were examined in Pb(II)-hypercontracted aortic rings. The authors observed that Pb(II) induced hypercontraction through the depletion of NO and by increased reactive oxygen species (ROS). Eugenol promoted relaxation of Pb(II) hypercontracted aortic rings by increased NO bioavailability. This effect was probably mediated by the antioxidant proprieties of this compound. Peixoto-Neves et al. [63] showed that Eugenol promoted a concentration-dependent dilation of the cerebral arteries by an inhibition of Calcium voltage-dependent channel, which was confirmed by patch-clamp studies.

### 2.10. 1-Nitro-phenylethane

The 1-nitro-phenylethane (NP) is the first nitro compound isolated from essential oil, and one of the few natural products containing this functional group [64]. The cardiovascular effect of this compound, isolated from *A. canelilla* essential oil and others plants, was tested *in vivo* by Interaminense et al. [65]. NP promoted bradycardic and hypotensive responses after a bolus injection. That response was completely eradicated after cervical bivagotomy. The tests that were made with capsaicin and methylatropine suggested that the mechanism of action used by NP was mediated by both a vagal reflex and a cholinergic mechanism. Additionally, the application of NP directly on the heart shows that an effect of pulmonary C-fibers could also be involved.

### 2.11. Auraptene

Auraptene is a coumarin contained in the peels of citrus fruits such as *Citrus paradise* [66]. Razavi et al. [67] studied the effect of the chronic administration of auraptene on heart rate and blood pressure after eight weeks of treatment of normo- and hypertensive rats using the DOCA-Salt model of hypertension. They observed a dose- and time-dependent decrease in blood pressure in hypertensive rats and no changes in heart rate. While promising, the study failed to provide mechanisms since a biomolecular study was not performed.

### 2.12. Citral

Citral is a mixture of the isomeric aldehydes geranial and neral, which occurs in plants and citrus fruits. Pereira et al. [68] used aortic rings to investigate the vasorelaxant proprieties of this monoterpene. The authors showed that this compound promoted vasodilation in phenylephrine pre-contracted rings using an endothelium-independent mechanism. The results suggested that citral promoted relaxation by changing calcium dynamics, which is an effect that occurs once citral-inhibited contractions by both high K^+^ and phenylephrine manifest. However, the study did not investigate the effects of citral on calcium receptors.

### 2.13. Citronellal

The monoterpene citronellal is a major component of the essential oils in various aromatic species, such as *Cymbopogon winterianus* Jowitt (Java citronella), *Corymbia citriodora* (Hook.) K.D. Hill, and *C. nardus* L. [69]. Cardiovascular properties of citronellal were evaluated in an NO-inhibition model of experimental hypertension. Mean arterial pressure (MAP) was reduced by oral acute citronellal administration. Citronellal also induced vasorelaxation of the mesenteric arteries of rats using an endothelium-independent mechanism. Although the exact mechanism of the vasodilatory action of citronellal was not conclusive, it is possible to state that the hypotensive effect should be related to its vasodilatory action.

### 2.14. Farnesene

Farnesene is one of the major compounds of the German essential oil chamomile. The action of this sesquiterpene on vascular tone was investigated using porcine coronary and splenic arteries. It was demonstrated that farnesene did not promote a vasorelaxant effect even when concentrations up to 30 μM were used [51].

### 2.15. Limonene

Limonene is a monoterpene found in citrus fruits, especially in orange and lemon, with high concentration in their essential oils [70,71]. De Sousa et al. [61] investigated the effects of limonene in guinea pig tracheas and rat aortas. The authors observed that the compound produced relaxant effects on the tracheas and aortic rings independent of the endothelium. In this study, both (+)-limonene and (−)-limonene enantiomers were used, and no differences were observed. This indicated that limonene could promote antispasmodic effects on the smooth muscle of the trachea.

### 2.16. Linalool

The monoterpene linalool (LO) is the major constituent (87.7%) of Rosewood Oil (EOAR). De Siqueira et al. [72] observed that EOAR induced hypotension and bradycardia in awakened animals that were abolished by pre-treatment with methylatropine. Additionally, dose-dependent EOAR blunted the phenylephrine-induced contraction of the aortic rings. Isolated LO reduced the hypercontraction of aortic segments induced by heavy metals, such as As and Hg, and involved the enhancement of NO synthesis and the blockade of voltage-dependent calcium channels [60]. Deep investigation of the mechanism of action of LO indicated further participation of soluble guanylyl cyclase and K^+^ channel on its vasorelaxant effects [73].

### 2.17. Linalyl Acetate

The monoterpene ester linalyl acetate (LA) mobilized the intracellular calcium concentration ([Ca]i) in cultured vascular endothelial (EC) or in mouse vascular smooth muscle (MOVAS) cells. In EC, LA induced a transient increase followed by a sustained decrease in [Ca]i, whereas in MOVAS the increase remained unchanged. LA blocks the extracellular calcium influx in EC, but not in MOVAS. Therefore, LA differently affects the endothelium and smooth muscle cells and its effect on EC may explain its protective effect against endothelium dysfunction associated with cardiovascular diseases [74].

### 2.18. Menthol

Menthol is a monoterpene alcohol found in *Mentha* species such as *M. piperita* and *M. arvensis* [75]. Cheang et al. [76] used different arterial segments (aortae, coronary and mesenteric) to investigate the proprieties of the menthol to produce vasorelaxation. This monoterpene produced concentration-dependent and endothelium independent vasorelaxation. The contraction induced by CaCl_2_ was suppressed by menthol in a similar way as nifedipine (an L-type Ca^2+^ channel inhibitor) and indicated that menthol could affect this kind of channel. This study demonstrated that the vasorelaxant proprieties of menthol were not affected by cGMP or NO. Therefore, the vasorelaxation induced by menthol was probably caused by direct action on calcium dynamics.

### 2.19. N-Butylidenephthalide

*N*-Butylidenephthalide (BP) is obtained from the volatile oil of *Angelica sinensis* [77]. The effects of this compound in angiogenesis and cell proliferation *in vitro* and ex *vivo* were evaluated by Yeh et al. [77]. The researchers showed that this compound was capable of a concentration-dependent inhibition of endothelial proliferation, endothelial wound healing, and endothelial tube formation on human umbilical vein endothelial cells (HUVEC). The researchers also investigated the mechanism of action and determined that BP promoted apoptosis and an increased maintenance of the cell cycle during the G0–G1 phase. Additionally, BP decreased the capillary sprouting of the aorta and vascularization of zebrafish. Together, these results indicate the anti-angiogenic effect of BP.

### 2.20. Rotundifolone

Several studies using the essential oil of *Mentha x villosa* showed its hypotensive effect in a dose-dependent manner in hypertensive rats (DOCA-salt hypertensive rats), which is probably associated with a vasorelaxative activity [78,79]. Similarly, hypotensive properties also were observed with the monoterpene rotundifolone, the main constituent of the essential oil of *Mentha x villosa*, comprising approximately 63% of its content. Rotundifolone promotes a concentration-dependent vasorelaxant effect on the superior mesenteric arteries of rats. The mechanism by which rotundifolone promoted this relaxation was investigated using patch-clamp techniques. The experiment demonstrated that rotundifolone probably acted by calcium-activated potassium (BK_Ca_) channel activation, once tetraethylammonium (TEA) eradicated the observed relaxation. However, this mechanism of action seemed to be more important at low doses and on conducting arteries (aorta). Additionally, changes on the L-type Ca^2+^ channels at high concentrations could also precipitate the mechanism by which rotundifolone caused vascular relaxation.

### 2.21. α-Terpineol

The *in vivo* effect of the monoterpene α-terpineol was evaluated by Sabino et al. [80] who used the model of hypertension induced by l-NAME. Therefore, it was demonstrated that α-terpineol was capable of inducing a dose-dependent hypotension in awake animals. Likewise, when mesenteric rings were used, the researchers showed that this compound produced vasorelaxant effects independent of the endothelium. Those vasorelaxant effects could be attributed, at least in part, to the inhibition of voltage-dependent calcium channels. Additionally, treatment with α-terpineol for seven days promoted an induced antioxidant effect in the animals treated with l-NAME. The values of SOD, catalase and glutathione peroxidase activity were restored to values similar to the control groups, which indicated the antioxidant capacity of α-terpineol.

The *in vitro* and *in vivo* cardiovascular effects of the chemical constituents of essential oils are shown in Table 1 and Table 2.

### 2.22. 1,8-Cineole

The effect of cyclic monoterpene 1,8-cineole was investigated on systolic blood pressure (SBP) and oxidative stress in rats chronically exposed to nicotine. The dose of 0.1 mg/kg of this monoterpene significantly reduced SBP, while, at the dose of 1.0 mg/kg, there was an increase of plasma nitrite concentrations. At doses of 0.01 mg/kg and 0.1 mg/kg, 1,8-cineole also antagonized nicotine-induced lipid peroxidation. It has been suggested that the regulation of nitric oxide and oxidative stress in rats should contribute to the antihypertensive effect of 1,8-cineole [83].

## 3. Conclusions

Essential oils are a new option of bioactive substances in animal models that are being used in the study of new candidates for cardiovascular drugs. Due to the diversity of chemical structures and mechanisms of action, such as blockade of voltage dependent L-type Ca^2+^ channels, cholinergic and vagal reflex activation, and participation of muscarinic receptors, it is not possible to establish a principal chemical characteristic of cardiovascular activity. The lipid solubility and volatility are common properties of these substances. However, several compounds have high pharmacological potency, for example 1,8-cineole and borneol. Pharmacological evaluation of the synthetic derivatives of these constituents appears to be an interesting way to optimize the pharmacologic profile and advance the knowledge to promote accomplishing prototypes for new cardiovascular drugs. In addition, the simplicity of the chemical structures of the bioactive compounds may result in the preparation of low cost drug candidates.

## Figures and Tables

**Table 1:** *In vitro* cardiovascular effects of the chemical constituents of essential oils.

Compound	Assay	Concentration	Effects	Reference
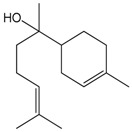 **Bisabolol**	Rat smooth muscle from vascular and non-vascular	1–1000 μmol/L	Relaxation by acting in Ca^2+^ voltagem-dependent channel	[49]
	Porcine splenic artery and coronary artery	3, 10 and 30 μM	Vasodilatation by inhibiting calcium influx	[51]
	Rat thoracic aorta and mesenteric ring	1–1000 μmol/L	Vasorelaxation by acting in Ca^2+^ voltagem-dependent channel	[50]
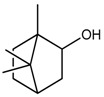 **Borneol**	Human internal mammary artery	Phytotherapic preparation enriched with borneol (1 mg/mL)	Vasorelaxation with and without endothelium	[56]
	Rat thoracic aorta artery	10^−9^ to 3 × 10^−4^ M	Vasorelaxant effect, probably by potassium channels activation, reduction in calcium influx and inhibition of calcium mobilization from intracellular stores	[55]
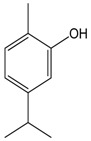 **Carvacrol**	Rat thoracic aorta artery	1, 10 and 100 μmol L^−1^	Vasorelaxant effect via inhibition of ROS and stimulation of NOS	[52]
	Rat Cerebral and cerebellar pial Arteries	10 and 30 μM	Vasodilatation by increase on calcium influx, by activating TRPV3 channel	[13]
	Rat superior mesenteric artery	10^−8^ to 3 × 10^−4^ M	Vasorelaxation by inhibition calcium influx through the L-type Cav, ROC and SOC channels	[53]
	Atria isolates	10 μM and 100 μM	Negative inotropic and chronotropic effect	[53]
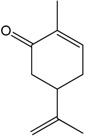 **Carvone**	Rat thoracic aorta	100 μM	Vasorelaxation by blocking calcium influx through VDDC	[60]
	Rat thoracic aorta and trachea of guinea pigs	10^−6^ to 3 × 10^−4^ M	Vasorelaxant effect	[61]
	Rat thoracic aorta	10^−7^, 10^−6^, 10^−5^, and 10^−4^ g/mL	Vasodilatory effect by inhibiting both Ca^2+^ influx and Ca^2+^ release	[43]
	Porcine coronary artery	32–320 μM	Vasorelaxation by inhibiting Ca^2+^ sensitivity and Ca^2+^ influx	[42]
	Ventricular cardiomyocytes and vascular smooth muscle cells	0.01–1000 μM	Vasorelaxing action by inhibiting L-type Ca^2+^ channels and possible participation of TRPA1	[44]
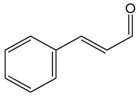 **Cinnamaldehyde**	Rat aorta artery and Human	10 μM	Prevents endothelial dysfunction by attenuating ROS generation and	
	umbilical vein endothelial cells (HUVECs)		preserving nitric oxide levels and Nrf2 activation and the up-regulation of downstream target proteins	[40]
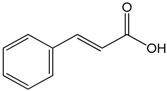 **Cinnamic acid**	Rat thoracic Aorta	0.1 mM, 0.2 mM, 0.4 mM, 1 mM, and 2 mM	Vasodilation via the NO–cGMP-PKG pathway, which stimulates Ca^2+^-activated K^+^ channels	[46]
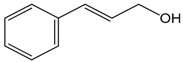 **Cinnamyl alcohol**	Rat thoracic aorta artery	0.2 mM, 0.4 mM, 0.6 mM, 1 mM or 1.5 mM	Vasodilation by activation of K^+^ channels and inhibition of Rho-kinase, which inhibit Ca^2+^ sensitization via the NO-cGMP-PKG pathway	[47]
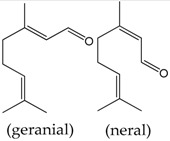 **Citral (= geranial + neral)**	Rat thoracic Aorta artery	0.6 to 6 mM	Vasorelaxation by reduced the calcium influx by the blockade of voltage dependent L-type Ca^2+^ channels	[68]
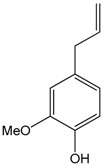 **Eugenol**	Rat atria Muscle	1, 3, 5, 7, and 10 mM	Increase in resting tension by cooperative activation of cardiac thin filaments by strongly attached cross-bridges (rigor state)	[63]
	Rat atria Muscle	1, 3, 5, 7, and 10 mM	Increase in resting tension by cooperative activation of cardiac thin filaments by strongly attached cross-bridges (rigor state)	
	Rat thoracic aorta artery	1, 10 and 100 μmol L^−1^	Vasorelaxant effect via inhibition of ROS and stimulation of NOS	[52]
	Rat thoracic aorta artery	100 μM	Vasorelaxation by inhibiting ROS and elevating NO	[60]
	Rat cerebral artery	100 μM	Vasorelaxation by inhibiting voltage-dependent Ca^2+^	[63]
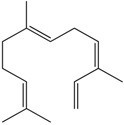 **Farnesene**	Porcine splenic artery and coronary artery	3, 10 and 30 μM	Vasodilatation by inhibiting calcium influx	[51]
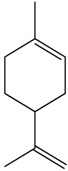 **Limonene**	Rat thoracic aorta and trachea of guinea pigs	10^−6^ to 3 × 10^−4^ M	Vasorelaxant effect	[61]
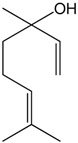 **Linalool**	Rat thoracic aorta	500 μM	Vasorelaxation by activating sGC and K^+^ channels and by inhibiting Ca^2+^ influx	[73]
	Rat thoracic aorta	100 μM	Vasorelaxation by blocking voltage dependent calcium channel (VDCC) and elevating NO	[60]
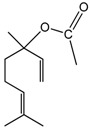 **Linalyl acetate**	The mouse vascular smooth muscle cell line MOVAS-1 (MOVAS) and human umbilical vein endothelial cell line EA.hy926 (EA)	0.01% *v*/*v*	Increase the intracellular K^+^ levels	[74]
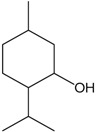 **Menthol**	Rat aorta, mesenteric and coronary arteries	0.01-1 mM	Vasorelaxation through inhibiting Ca^2+^ influx via nifedipine-sensitive Ca^2+^ channels in vascular smooth muscle	[76]
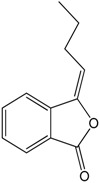 ***N*-Butylidenephtalide**	Human umbilical vein endothelial cells (HUVECs)	20–50 μg/mL	Anti-angiogenic activities by increase of maintaining cell cycle on G0–G1 phase, and promoting apoptosis	[77]
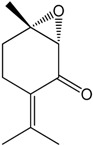 **Rotundifolone**	Rat superior mesenteric artery	10^−7^ to 3 × 10^−3^ M	Vasorelaxation through activation of BK_Ca_ channels and by the inhibition of Ca^2+^ entry through L-type Ca^2+^ channels	[81]

**Table 2:** *In vivo* cardiovascular effects of the chemical constituents of essential oils.

Compound	Model	Dose	Effects	Reference
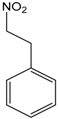 **1-Nitro-phenylethane**	Spontaneously hypertensive rats (SHR)	1–10 mg/kg	Promotes bradycardic and hypotensive responses after in bolus application. The mechanism suggested is by cholinergic and vagal reflex activation	[65]
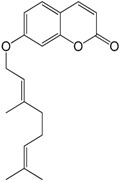 **Auraptene**	DOCA-salt hypertensive rats	2–16 mg/kg/day	Decrease of blood pressure dose and time dependent	[67]
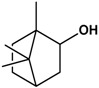 **Borneol**	Transient cerebral ischemia by intraluminal middle cerebral artery occlusion (MCAO)	0.8 mg/kg	Decrease of pro-inflammatory markers and infarct area. Antioxidant proprieties of borneol were associated with these effects	[57]
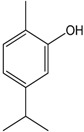 **Carvacrol**	Wistar rats	1–20 mg/kg	Hypotension and bradycardic effects associated with a decrease in heart rate, in a dose-dependent manner	[53]
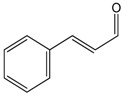 **Cinnamaldehyde**	Isoproterenol model of myocardial ischemia	22.5, 45 and 90 mg/kg	Decrease of cardiac injury and pro-inflammatory cytokines after per-treatment (14 days), additionally an increase of NO and SOD levels of heart tissue	[14]
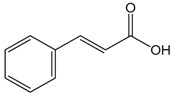 **Cinnamic acid**	Isoproterenol model of myocardial ischemia	37.5, 75 and 150 mg/kg	Decrease of biochemical markers of myocardial infarct and increase of NO levels. Indicating antioxidant proprieties of CD	[14]
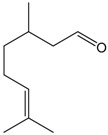 **Citronellal**	l-NAME hypertensive and normotensive rats	5, 10, 20, and 40 mg/kg in bolus and 200 mg/kg orally	In bolus promotes hypotensive and bradycardic effects of normotensive rats. Treatment of l-NAME hypertensive rats promotes decrease of MAP. The results suggest that muscarinic receptors could be involved	[82]
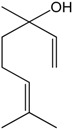 **Linalool**	Normotensive rat	1–20 mg/kg	They evaluate the essential oil of Rosewood enriched with linalool. *In bolus* treatment promotes biphasic hypotension and bradycardic responses by vagal reflex and cholinergic mechanism	[72]
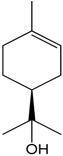 **α-Terpineol**	l-NAME hypertensive rats	25–100 mg/kg/day	Cardioprotective effect by induced hypotension and antioxidant potential by restoring antioxidant enzyme activities (catalase and glutathione peroxidase)	[80]
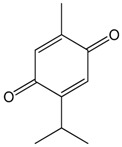 **Thymoquinone**	Airway inflammation by acute exposure to diesel exhaust particles (DEP)	0.01–0.1 mg/mL	Pre-treatment with thymoquinone prevents the worse effects promoted by DEP such as leukocytosis, increase of IL-6 and decrease of SOD plasma activity. The platelet numbers and prothrombotic events were also decreased	[34]
	Isoproterenol model of myocardial ischemia	12.5–50 mg/kg	Antioxidant and Cardioprotective effects by decrease of LDH levels and TBARS activity. The SOD activity was increased to almost normal levels. The GSH/GSSG ratio decreased gradually and returned to near normal levels with corresponding increases in the dose	[35]
	Abdominal aorta ischemia followed by reperfusion (I/R)	20 mg/kg	Reduction of oxidative stress determined by Total Oxidant Status and Oxidative Stress Index in blood samples. Decreased of histopathologic injury in in lung, renal, and heart tissues	[33]
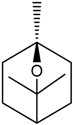 **1,8-Cineole**	Systolic blood pressure measured in rats	0.1 mg/kg	Antihypertensive activity associated with the regulation of nitric oxide and oxidative stress in rats chronically exposed to nicotine	[83]

## Abbreviations

AID	Alfa (α) Interaction Domain
BP	*N*-Butylidenephthalide
BS	(−)-α-Bisabolol
CAL	Cinnamyl Alcohol
Cavβ2a	β-Subunit isoform of voltage-gated L-type Ca^2+^ channel
cGMP	Cyclic *guanosine* monophosphate
CK-MB	Creatine kinase MB
CVD	Cardivascular diseases
DOCA	Deoxycorticosterone acetate
EC	Cultured vascular endothelial
EDHF	Endothelium-Derived Hyperpolarizing Factor
eNOS	Endothelial NO Synthase
EOAR	Rosewood Oil
GR	Glutathione Reductase
GSH/GSSG	Reduced or Oxidized Glutathione ratio
HUVEC	Human Umbilical Vein Endothelial Cells
IK	Active Intermediate
IL-1β	Interleukin 1β
IL-6	Interleukin 6
iNOS	Inducible NO Synthase
LA	Linalyl Acetate
LDH	Lactate Dehydrogenase
l-NAME	*N*(ω)-Nitro-l-Arginine Methyl Ester
LO	Linalool
MAP	Mean Arterial Pressure
MI	Myocardial Injury
MOVAS	Mouse Vascular Smooth Muscle
NCD	Non Communicable Diseases
NO	Nitric oxide
NP	1-Nitro-Phenylethane
Nrf2	NF-E2-related factor 2
PKG	Protein Kinase G
RAS	Renin-angiotensin Aldosterone System
ROC	Receptor Operator Channels
ROS	Reactive Oxygen
SBP	Species Systolic Blood Pressure
SK	Small-conductance Ca^2+^-activated K^+^
SKCa/IKCa	Calcium Activated Potassium Channels
SOCs	Store Operator Channels
SOD	Superoxide Dismutase
TBARS	Thiobarbituric Acid Reactive Substances
TEA	Tetraethylammonium
TNF-α	Tumor Necrosis Factor alpha
TQ	Thymoquinone
TRPA1	Transient Receptor Potential Ankyrin 1
TRPV	Transient Receptor Potential Vanilloid
VCM	Ventricular Cardiac Myocytes
VOCC	Voltage-Operated Calcium Channel
VSMC	Vascular Smooth Muscle Cells
